# The Inverse Correlation Between the Duration of Lifetime Occupational Radiation Exposure and the Prevalence of Atrial Arrhythmia

**DOI:** 10.3389/fcvm.2022.863939

**Published:** 2022-05-30

**Authors:** Rithika Thirumal, Catherine Vanchiere, Ruchi Bhandari, Sania Jiwani, Ronald Horswell, San Chu, Surbhi Chamaria, Pavan Katikaneni, Marjan Boerma, Rakesh Gopinathannair, Brian Olshansky, Steven Bailey, Paari Dominic

**Affiliations:** ^1^Department of Internal Medicine, University of Cincinnati, Cincinnati, OH, United States; ^2^School of Medicine, Louisiana State University Health Sciences Center, Shreveport, LA, United States; ^3^Department of Internal Medicine, Temple University, Philadelphia, PA, United States; ^4^Department of Epidemiology and Biostatistics, West Virginia University, Morgantown, WV, United States; ^5^Department of Internal Medicine, Louisiana State University Health Sciences Center, Shreveport, LA, United States; ^6^Pennington Biomedical Research Center, Louisiana State University, Baton Rouge, LA, United States; ^7^Mercy Hospital, Fort Smith, AK, United States; ^8^Center for Cardiovascular Diseases and Sciences, Louisiana State University Health Sciences Center, Shreveport, LA, United States; ^9^Department of Pharmaceutical Sciences, University of Arkansas Medical Center, Little Rock, AK, United States; ^10^Department of Cardiology, Kansas City Heart Rhythm Institute, Overland Park, KS, United States; ^11^Department of Medicine, University of Iowa Hospitals and Clinics, Iowa City, IA, United States

**Keywords:** atrial arrhythmia, fluoroscopy, radiation, interventional cardiologists, electrophysiologists, invasive cardiology

## Abstract

**Objective:**

Advancements in fluoroscopy-assisted procedures have increased radiation exposure among cardiologists. Radiation has been linked to cardiovascular complications but its effect on cardiac rhythm, specifically, is underexplored.

**Methods:**

Demographic, social, occupational, and medical history information was collected from board-certified cardiologists via an electronic survey. Bivariate and multivariable logistic regression analyses were performed to assess the risk of atrial arrhythmias (AA).

**Results:**

We received 1,478 responses (8.8% response rate) from cardiologists, of whom 85.4% were male, and 66.1% were ≤65 years of age. Approximately 36% were interventional cardiologists and 16% were electrophysiologists. Cardiologists > 50 years of age, with > 10,000 hours (h) of radiation exposure, had a significantly lower prevalence of AA vs. those with ≤10,000 h (11.1% vs. 16.7%, *p* = 0.019). A multivariable logistic regression was performed and among cardiologists > 50 years of age, exposure to > 10,000 radiation hours was significantly associated with a lower likelihood of AA, after adjusting for age, sex, diabetes mellitus, hypertension, and obstructive sleep apnea (adjusted OR 0.57; 95% CI 0.38–0.85, *p* = 0.007). The traditional risk factors for AA (age, sex, hypertension, diabetes mellitus, and obstructive sleep apnea) correlated positively with AA in our data set. Cataracts, a well-established complication of radiation exposure, were more prevalent in those exposed to > 10,000 h of radiation vs. those exposed to ≤10,000 h of radiation, validating the dependent (AA) and independent variables (radiation exposure), respectively.

**Conclusion:**

AA prevalence may be inversely associated with radiation exposure in Cardiologists based on self-reported data on diagnosis and radiation hours. Large-scale prospective studies are needed to validate these findings.

## Introduction

The field of cardiology has made remarkable progress in the past 30 years in the development of percutaneous therapies. These new catheterization procedures have lead to increased radiation exposure among cardiologists which has become an area of concern (1). Fluoroscopic procedures are a source of medical occupation related radiation exposure and, with cardiac procedures becoming more intricate, radiation exposure has increased, further amplifying the need to investigate the impact of occupational radiation exposure (2–4).

Atrial tachycardia (AT), atrial flutter (Afl), and atrial fibrillation (AF) are a continuum of atrial arrhythmias (AA) that lead to atrial remodeling and increased risk of thrombo-embolism (5, 6). Atrial fibrillation (AF) is a common clinical arrhythmia that increases the risk of stroke, heart failure, and other cardiac complications (7, 8). Many risk factors have been established for AA, including hypertension (HTN), diabetes mellitus (DM), obstructive sleep apnea (OSA), coronary artery disease (CAD), and advanced age. Recently, high dose radiation therapy, as seen in cancer patients, has been associated with an increased incidence of AF. This may be due to oxidative DNA damage leading to cardiac inflammation and fibrosis, both of which are risk factors for AF (7, 9–11). Ironically, while ablative radiation with stereotactic body radiation therapy can be therapeutically useful for patients with ventricular arrythmias, exposure to longitudinal low-dose radiation can be detrimental to the operators (12, 13).

Although cardiologists are not exposed to high acute doses of radiation, their longitudinal exposure to low doses is cause for concern, particularly as the arrhythmogenic effects of this exposure are still unclear. Studies have surveyed invasive cardiologists to assess a variety of occupational hazards, including orthopedic and ophthalmologic complications, but the impact of occupational radiation exposure on the prevalence of AA is still unexplored in this cohort (11, 14, 15).

In this study, we examined the impact of cumulative low-dose radiation exposure on cardiovascular health and, more specifically, the risk of AA among cardiologists exposed to procedural radiation. We hypothesize that increased radiation exposure is associated with a higher prevalence of AA. The results of this study can help guide future research and increase awareness of the impact of radiation exposure in the field of invasive cardiology.

## Methods

An electronic survey was distributed to 16,790 board-certified cardiologists (~42% were interventional cardiologists or electrophysiologists) who were members of the American College of Cardiology, the Society of Cardiovascular Angiography and Interventions, or the Heart Rhythm Society (16). Along with the survey link (Supplementary Material 1), the participants received a brief description of the study and a consent letter that outlined the potential risks involved with participation in the study. Participants were also asked to distribute the survey to colleagues in their practices and/or institutions to maximize the number of potential responses. As the initial step, participants were informed that continuing to the survey serves as their consent for study participation; no written consent was obtained. Participants completed a brief survey collecting information regarding elements of their demographic, social, occupational, and medical health histories. No identifiers were obtained during data collection. Follow-up email reminders were sent over a period of 4 months to maximize the number of responses (Supplementary Figure 1). All de-identified survey submissions were included in the analysis. The research reported in this article was approved by the Louisiana State University institutional review board and adheres to institutional guidelines.

### Statistical Analysis

For this study, the outcome variable, AA, was defined as one or more of the following: frequent premature atrial contractions, AT, AFL, and AF. The diagnosis of AA was self-reported, included symptomatic and asymptomatic clinical diagnosis and were not restricted to any particular mode of diagnosis. The key predictor variable, total hours of radiation exposure, was calculated by multiplying “approximate hours of occupational radiation exposure per week” by 52 and “years of occupational radiation exposure.”

We compared the likelihood of AA among cardiologists with significant radiation exposure to minimally radiated cardiologists, and, in the process, determined the impact of occupational radiation exposure on cardiovascular health. Frequencies and percentages are presented for the following groups: (1) prevalence of comorbidities among those with and without AA > 50 years of age; (2) demographic, social, and occupational characteristics among those with 10,000 h or less of radiation exposure versus greater than 10,000 h; (3) prevalence of comorbidities among those with 10,000 h or less of radiation exposure versus greater than 10,000 h; (4) demographic, social, and occupational characteristics among all cardiologists vs. those with AA; (5) prevalence of AA based on procedure performed. Each of these groups was compared with Chi-square test or Fisher's exact test when expected cell count was <5. Bivariate and multivariable logistic regression analyses were conducted to determine the association of AA with key demographic factors, comorbidities and total hours of radiation exposure. Results were considered statistically significant at *p* < 0.05. All analyses were conducted in SPSS version 27.0 (17).

## Results

### Characteristics of Respondents

**Figure 1:**
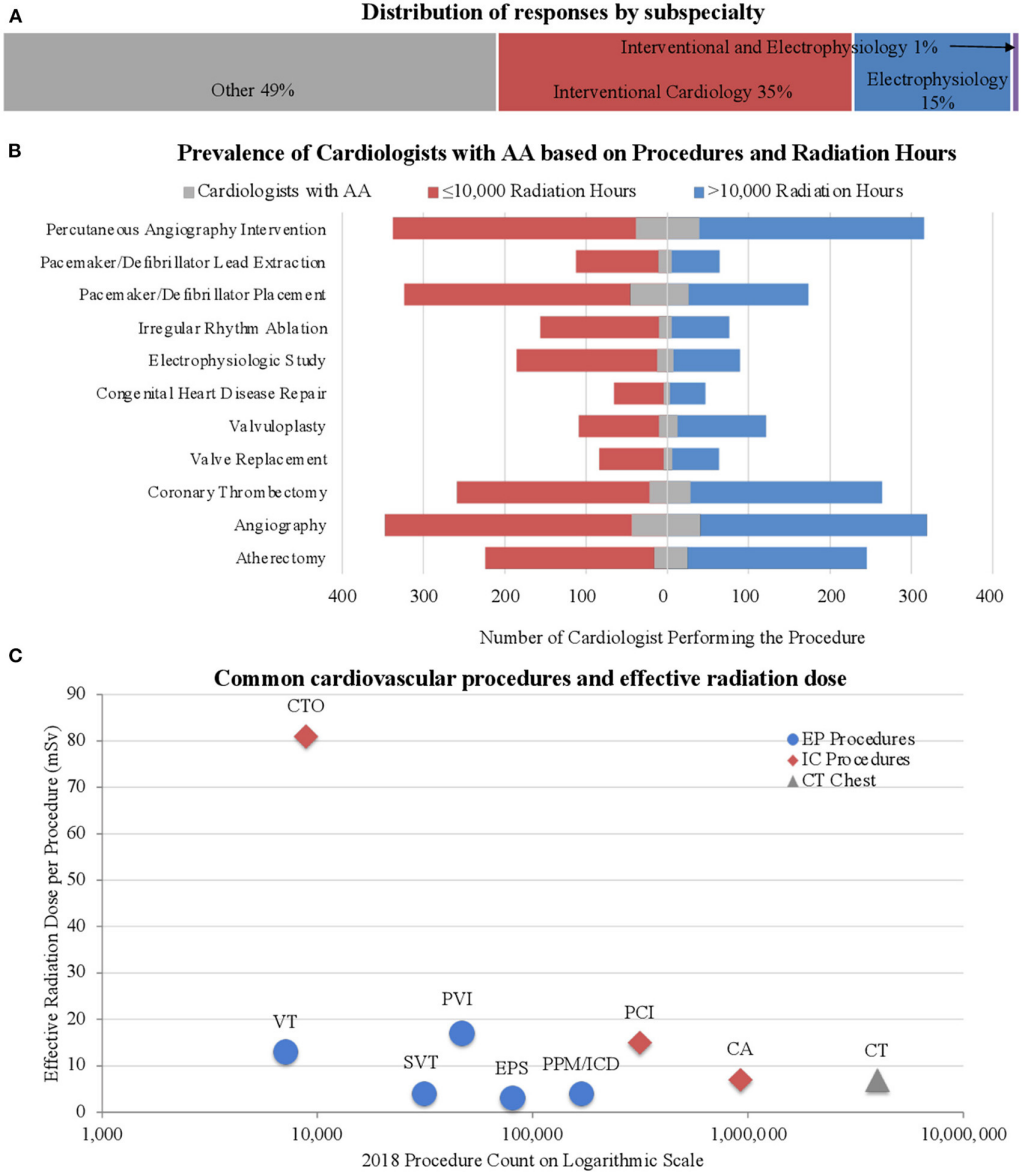
Subspecialty and procedural distribution. **(A)** Distribution of responses based on subspecialty. Approximately half the respondents were invasive cardiologists. **(B)** Prevalence of AA among cardiologists based on type of procedure. Note the increased occurrence of AA among those with lower radiation exposure, especially in those performing pacemaker/defibrillator placement. **(C)** The number of procedures performed in the United States was collected from the 2018 CMS Part B National Summary Data File (18). The effective dose of radiation per procedure was determined through a review of the literature (19, 20). Our survey participants reported procedure frequencies consistent with those found nationwide from the 2018 data as described. The chest CT is depicted as a reference value. CTO, Chronic Total Occlusion; VT, VT ablation; SVT, SVT ablation; PVI, Pulmonary vein Isolation; EPS, EP Study; PPM/ICD, Pacemaker and ICD Placement; PCI, Percutaneous Coronary Intervention; CA, Coronary Angiography; CT, Chest CT (Reference).

The survey was completed by 1,478 cardiologists (8.8% response rate); ~10% response rate among interventional cardiologists and electrophysiologists and 7% response rate among other subspecialties (16). The respondents were predominately male (86.1%) and white/Caucasian (79.0%). The median age was 56–60 years; two-thirds were ≥65 years of age (Supplementary Table 1). Interventional cardiologists constituted 35.6% of the respondents and 16.4% were electrophysiologists (Figure 1A). A chest/abdomen protective lead attire (vest or apron) was always worn by 73.3% of invasive cardiologists (interventional cardiologists and electrophysiologists). As outlined in Supplementary Table 2 and Figure 1B, the procedures most performed by the survey participants were angiography (45.1%), percutaneous coronary interventions (44.1%), coronary thrombectomy (35.4%), and pacemaker/defibrillator placement (33.6%). Pulsed fluoroscopy was the primary imaging modality, used by 44.2% of the respondents who used imaging, compared to 19.7 and 9.3% who used low and high dose cineangiography, respectively. Among their social habits, 74.7% of all participants consumed alcohol and 2.2% were current tobacco users. Various co-morbidities were observed among respondents (Supplementary Table 3), including AA (11.1%), cancer (11.3%), cataracts (18.1%), carotid artery disease (1.4%), CAD (8.7%), DM (5.5%), dyslipidemia (27.1%), HTN (30.4%), OSA (8.0%), and stroke/transient ischemic attack (TIA) (2.5%).

### Radiation Exposure and AA

**Figure 2:**
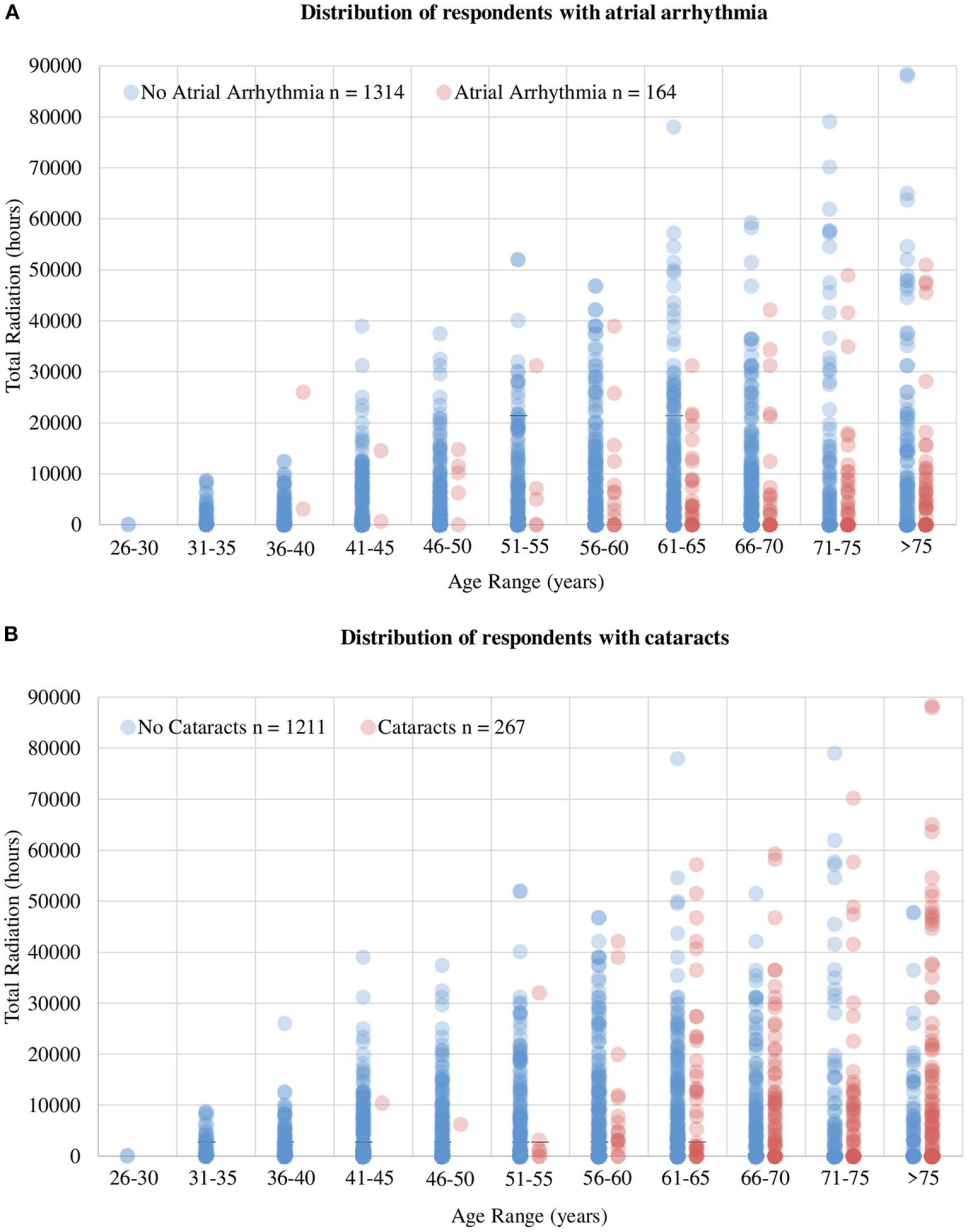
Distribution of respondents with AA and cataracts. **(A)** Total number of radiation hours per age group in cardiologists reporting AA and those without AA. It exhibits a clear delineation of number of cases of AA around 10,000 h of radiation and 50 years of age, leading to the thresholds used to stratify response during analysis. **(B)** The influence of age and total radiation hours on the prevalence of cataracts. The influence of increasing age on the prevalence of cataracts is evident but more importantly, note the increased prevalence of cataracts with increasing total hours of radiation, indicating the accuracy of the dataset collected as well as validating the selection of 10,000 radiation hours as the threshold to delineate exposure groups.

Aligning with the objective of the study, a focused analysis regarding the risk of AA was performed. Among the respondents, 9.2% had AF, 3.3% had frequent premature atrial contractions, 2.6% had Afl, and 1.6% had AT, leading to an AA prevalence of 11.1% among our participants. AA was significantly more prevalent among men than women (15.7 vs 5.4%, *p* = 0.004) and in those ≥65 years of age vs. those <65 years of age (21.4 vs. 8.7%, *p* < 0.001). The likelihood of AA was significantly lower among cardiologists performing any of the following procedures: atherectomy, electrophysiologic studies, irregular rhythm ablations, and pacemaker/defibrillator lead extractions (Supplementary Table 2, Figure 1B). There was an approximately two-fold increase in AA prevalence in the presence of the following risk factors: DM (23.2% AA in those with DM vs. 10.4% AA in those without DM, *p* < 0.001), CAD (34.9% vs 8.8%, *p* < 0.001), HTN (16.0% vs 8.9%, *p* < 0.001), and OSA (26.3% vs 9.8%, *p* < 0.001). To determine the association between radiation exposure and the risk of AA, we first examined the distribution of respondents with AA stratified by age groups, as a function of hours of exposure to radiation. We found a clear separation of the number of respondents reporting AA around 10,000 h of exposure (Figure 2A). As most cardiologists <50 years of age had <10,000 h of exposure to radiation, and the conditions being evaluated generally increase with age, we focused on cardiologists >50 years of age. To validate the threshold of 10,000 h of exposure to radiation to study its relationship to AA, another well-established, rarely disputed consequence of occupational radiation exposure among cardiologists, namely cataracts, was initially investigated. Figure 2B shows the increased manifestation of cataracts with age and with increasing hours of exposure to radiation. A multivariable regression analysis showed that apart from age, >10,000 h of radiation exposure was an independent risk factor for the likelihood of cataracts (adjusted OR 1.72; 95% CI 1.24–2.37, *p* = 0.001), in agreement with historical data. Based on this, further investigations regarding the influence of radiation exposure on the cardiovascular health of cardiologists was focused on the 1,033 participants who were >50 years of age (Supplementary Figure 1).

### Baseline Characteristics of Cardiologists > 50 Years of Age Based on Exposure Hours

Compared to those with low radiation exposure (≤10,000 h), those with high radiation exposure (> 10,000 h) were more likely to be male (93.4% males in high exposure group vs. 86.7% in low exposure group, *p* = 0.005) and were more likely to be interventional cardiologists/electrophysiologists rather than another sub-specialty (79.4 vs. 20.6%, *p* < 0.001, Table 1). Cardiologists with high radiation exposure were significantly more likely to perform the procedures outlined in Table 1. Alcohol consumption and tobacco use were similar in the two groups. Approximately 43% of cardiologists in the low radiation exposure group did not have any type of occupational radiation exposure. Most cardiologists' institutions monitor radiation exposure (71.2% cardiologists) via dosimetry; among these, only 3.6% of cardiologists had crossed their institution's threshold (4.6% in low exposure vs. 2.6% in high exposure, *p* = 0.15). Almost all of the cardiologists (99.1%) with high radiation exposure wore lead attire covering the thoracic/abdominal area (vest or apron); 12.3 and 3.7% wore protective attire covering the head and shins, respectively; and 87.5% of high radiation exposed cardiologists used a front shield during procedures.

**Table 1:** Demographics, social, and occupational history based on hours of radiation exposure.

		# of Cardiologists with ≤10,000 h (%) *n* = 648^*^	**# of Cardiologists with** > 10,000 h (%) *n* = 350^*^	***p*-value^†^**
**Demographic characteristics**				
Sex	Male	560 (86.7%)	325 (93.4%)	0.005
	Female	84 (13.0%)	22 (6.3%)	
Age	≥ 66 years	309 (47.7%)	160 (45.7%)	0.552
	51–65 years	339 (52.3)	190 (54.3)	
Race	White/Caucasian	568 (88.2%)	304 (87.6%)	0.453
	Black/African American	11 (1.7%)	3 (0.9%)	
	Other	65 (10.1%)	40 (11.5%)	
Ethnicity	Hispanic	15 (2.6%)	12 (3.6%)	0.355
**Social history**				
Hx of alcohol use		519 (80.6%)	279 (80.2%)	0.874
Hx of alcohol abuse		46 (7.2%)	14 (4.1%)	0.051
Hx of tobacco use	Present, current	12 (1.9%)	9 (2.6%)	0.750
	Absent, quit	578 (90.3%)	311 (89.4%)	
	Absent, never	50 (7.8%)	28 (8.0%)	
**Occupational history**				
Type of cardiologist	EP and interventional cardiology	194 (29.9%)	278 (79.4%)	<0.001
	Other	454 (70.1%)	72 (20.6%)	
Type of procedure	Atherectomy	90 (13.9%)	204 (58.3%)	<0.001
	Angioplasty	189 (29.2%)	272 (77.7%)	<0.001
	Coronary thrombectomy	120 (18.5%)	223 (63.7%)	<0.001
	Transcatheter valve replacement	20 (3.1%)	41 (11.7%)	<0.001
	Valvuloplasty	41 (6.3%)	93 (26.6%)	<0.001
	Congenital heart disease repair	27 (4.2%)	34 (9.7%)	<0.001
	Electrophysiologic study	97 (15.0%)	71 (20.3%)	0.032
	Irregular rhythm ablation	72 (11.1%)	58 (16.6%)	0.014
	Pacemaker/defibrillator placement	201 (31.0%)	148 (42.3%)	<0.001
	Pacemaker/defibrillator lead Extraction	51 (7.9%)	53 (15.1%)	<0.001
	Percutaneous angiographic intervention	180 (27.8%)	271 (77.4%)	<0.001
Imaging most used	Pulsed fluoroscopy	209 (32.4%)	209 (60.2%)	<0.001
	Low-frame cineangiography	93 (14.4%)	92 (26.5%)	
	High-frame cineangiography	66 (10.2%)	46 (13.3%)	
	None	277 (42.9%)	0 (0.0%)	
Dosimetry threshold	Crossed	17 (4.6%)	9 (2.6%)	0.150
	Never crossed	352 (95.4%)	338 (97.4%)	
Protective attire	Head cap	41 (6.3%)	43 (12.3%)	0.001
	Shin shields	5 (0.8%)	13 (3.7%)	0.001
	Front shield	287 (77.4%)	302 (87.5%)	<0.001
	Vest or apron	368 (56.8%)	347 (99.1%)	<0.001

* *Sample number may vary due to exclusion of respondents electing to not answer*.

† *Chi-squared test, p < 0.05 is considered significant*.

### Risk of AA in Cardiologists > 50 Years of Age Based on Total Hours of Radiation Exposure

Among cardiologists >50 years of age, the difference in the risk of AA based on specific procedures was not significant. However, when categorized by subspecialty, electrophysiologists and interventional cardiologists combined had a significantly lower prevalence of AA compared to other types of cardiologists (10.5 vs. 18.5%, *p* < 0.001). The effects of traditional risk factors on the prevalence of AA were assessed in cardiologists and found to be similar to those in the general population. There was an increased prevalence of DM, CAD, congestive heart failure (CHF), OSA, and valvular heart disease in cardiologists with AA compared to those without AA (Supplementary Table 3).

A significant decrease in AA was observed among cardiologists with high radiation exposure compared to those with low radiation exposure (11.1 vs. 16.7%, *p* = 0.019, Table 2). Apart from cataracts, which were more prevalent in those with high radiation exposure (30.9 vs. 23%, *p* = 0.007), other medical conditions, many of which are risk factors for AA, were equally distributed in the two groups, including cancer (15.3% in low exposure vs. 14.6% in high exposure, *p* = 0.766), CAD (13.6 vs. 10.3%, *p* = 0.132), DM (7.6 vs. 6.9%, *p* = 0.683), HTN (38.0 vs. 41.1%, *p* = 0.326), and OSA (9.7 vs. 11.7%, *p* = 0.326), indicating that these co-morbidities did not play a significant role in the difference in prevalence of AA in the low and high radiation exposure groups.

**Table 2:** Prevalence of medical conditions based on hours of radiation.

	**≤10,000 h prevalence (%) *n* = 648^**a**^**	**> 10,000 h prevalence (%) *n* = 350^**a**^**	***p*-value^**b**^**
**Medical condition**			
Atrial arrhythmia	108 (16.7%)	39 (11.1%)	0.019
Aortic atherosclerosis	23 (3.5%)	14 (4.0%)	0.719
Cancer	99 (15.3%)	51 (14.6%)	0.766
Cardiomyopathy	6 (0.9%)	5 (1.4%)	0.530
Carotid artery disease	14 (2.2%)	6 (1.7%)	0.631
Cataracts	149 (23.0%)	108 (30.9%)	0.007
Chronic obstructive pulmonary disease	7 (1.1%)	5 (1.4%)	0.762
Congestive heart failure	10 (1.5%)	1 (0.3%)	0.109
Coronary artery disease	88 (13.6%)	36 (10.3%)	0.132
Dermatitis	20 (3.1%)	16 (4.6%)	0.230
Diabetes mellitus	49 (7.6%)	24 (6.9%)	0.683
Dyslipidemia	215 (33.2%)	119 (34.0%)	0.793
Hypertension	246 (38.0%)	144 (41.1%)	0.326
Infertility	12 (1.9%)	8 (2.3%)	0.641
Ischemic heart disease	35 (5.4%)	14 (4.0%)	0.328
Myocarditis	3 (0.5%)	0 (0.0%)	0.556
Obstructive sleep apnea	63 (9.7%)	41 (11.7%)	0.326
Peripheral vascular disease	7 (1.1%)	4 (1.1%)	1.000
Pulmonary fibrosis	4 (0.6%)	0 (0.0%)	0.304
Pulmonary hypertension	2 (0.3%)	1 (0.3%)	1.000
Stroke/transient ischemic attack	20 (3.1%)	11 (3.1%)	0.961
Thyroid disease	51 (7.9%)	21 (6.0%)	0.276
Valvular heart disease	26 (4.0%)	15 (4.3%)	0.836

a *Sample number may vary due to exclusion of respondents electing to not answer*.

b *Chi-squared test, p < 0.05 is considered significant*.

A multivariable logistic regression was performed to identify the risk factors and protective factors of AA (Table 3) adjusting for age, sex, race, DM, HTN, and OSA. Apart from confirming the traditional risk factors of AA such as age, sex, and OSA, results also showed that exposure to >10,000 h of radiation was an independent protective factor against the risk of AA (adjusted OR 0.57; 95% CI 0.38–0.85, *p* = 0.007). The role played by personal radiation exposure, such as that received during CT scans and X-rays for personal healthcare, was also accounted for. There was a significantly lower prevalence of personal radiation exposure in the high occupational exposure group compared to low exposure group (81.6 vs. 88.2%, *p* = 0.002). As expected, there was a significantly higher prevalence of stroke/TIA in the setting of AA compared to without AA (5.9 vs. 2.8%, *p* = 0.049) but there was no significant difference of stroke/TIA among cardiologists based on hours of radiation (3.1% in low exposure vs. 3.1% in high exposure, *p* = 0.961).

**Table 3:** Odds of atrial arrhythmia in cardiologists.

	**Unadjusted OR (95% CI)^**a**^**	***p*-value^**b**^**	**Adjusted OR (95% CI)^**a**^**	***p*-value^**b**^**
**Atrial arrhythmia (>50 years)**				
Age	1.53 (1.36–1.72)	<0.001	1.49 (1.32–1.69)	<0.001
Sex	3.29 (1.41–7.58)	0.006	2.44 (1.20–5.81)	0.043
Race (Black/African American)	0.72 (0.16–3.17)	0.665	1.50 (0.32–7.04)	0.605
Diabetes mellitus	2.02 (1.15–3.54)	0.014	1.20 (0.65–2.21)	0.568
Hypertension	1.38 (0.97–1.95)	0.070	0.92 (0.63–1.35)	0.662
Obstructive sleep apnea	2.53 (1.60–4.02)	<0.001	2.04 (1.23–3.37)	0.006
>10,000 radiation hours	0.63 (0.42–0.93)	0.020	0.57 (0.38–0.86)	0.007

a *Odds ratio with 95% confidence interval*.

b *p < 0.05 is considered significant*.

## Discussion

Occupational radiation exposure in cardiologists may be associated with various complications. A few of these are well established, e.g., orthopedic complications and cataracts, while others are actively being investigated, e.g., infertility, but its effects on AA have not been previously explored (21). Historically, high-dose radiation (5,000 mSv) and moderate-dose radiation exposure (>500 mSv) were thought to have significant cardiovascular effects, such as stroke and heart disease; however, the degree of cardiovascular risk associated with lower doses (<500 mSv) is currently unknown (22–24). Invasive cardiologists, i.e., those exposed to chronic high levels of occupational radiation exposure, such as interventional cardiologists and electrophysiologists, are a unique population with consistent longitudinal exposure to low doses of radiation limited to the periphery. Although they are not exposed to the direct X-ray beam, invasive cardiologists receive radiation from the scatter, which can vary extremely, ranging from 0.04 to 38 μSv per procedure (25). Over the course of an interventional cardiologist's career, he or she is exposed to 50–200 mSv of ionizing radiation, which is equivalent to 2,500–10,000 chest X-rays (9, 12, 14, 15, 26). Newer techniques focus on minimizing, or even eliminating radiation use but these protocols are not widespread (27, 28). Most procedures still require some degree of radiation exposure. Using information gathered from the literature review (29), Figure 1C demonstrates the degree of radiation exposure based on the type of procedures performed and the number of each procedure performed per year. The number of each type of procedure performed nationwide is similar to those performed by our survey participants, providing additional validation of an accurate random sampling.

This study noted a self-reported lower prevalence of AA in cardiologists >50 years of age with >10,000 h of radiation exposure compared to cardiologists >50 years of age with ≤10,000 h of radiation exposure. Electrophysiologists and interventional cardiologists combined had a significantly lower prevalence of AA compared to other types of cardiologists (10.5 vs. 18.5%, *p* < 0.001). A multivariable regression analysis was performed to adjust for well-established risk factors of AA such as age, sex, OSA, HTN and DM, and >10,000 h of radiation was statistically less likely to be associated with AA when compared to ≤10,000 h of radiation. Well established risk factors of AA, such as age, male sex, HTN, DM, OSA, were still found to be significant risk factors within our data set in univariate analysis, internally validating our dependent variable (AA). In addition, the most studied and widely accepted consequence of occupational radiation exposure, cataracts, was significantly more prevalent among those with high radiation exposure compared to those with low radiation exposure, validating the independent variable (radiation exposure) as well. The prevalence of co-morbidities such as CAD, HTN, DM, and OSA were not significantly different between the low and high radiation exposure groups, further solidifying the evidence that the difference in likelihood of AA is dependent on a secondary factor, possibly their occupational radiation exposure. Given the design of this study, these findings should be interpreted with caution, however, it lays the foundation for further investigation.

The general benefits of low-dose radiation have been studied before but the specifics of the mechanism by which low doses of radiation might be protective against AA is limited and most likely multifactorial. A few theories can provide a possible explanation for this paradoxical finding, all of which may vary in significance.

Systemic inflammatory mechanisms have been implicated in the initiation and progression of arrhythmias, especially AF (30–32). Inflammation causes variation in membrane potential and ion channel disturbances, leading to cardiac fibrosis, which is a prominent substrate for arrhythmias (30, 31). Several controlled studies have shown increased levels of inflammatory markers in patients with AF compared to those in sinus rhythm (30, 31, 33). Low doses of ionizing radiation have anti-inflammatory effects through various mechanisms at the molecular level (34–38). These mechanisms may depress inflammation in cardiac tissue leading to a decrease in the likelihood of AA among cardiologists exposed to increased cumulative levels of low-dose radiation. However, prior studies were mostly performed with a single dose of radiation. Future studies should identify whether low doses of radiation experienced over a prolonged period have similar consequences of inflammation.

A recent mechanism implicated in AA progression, especially in AF, is DNA damage induced metabolic remodeling of cardiac tissue (39). Considering this finding, the cytogenetic adaptive response can elucidate the benefits of low-dose radiation in protection against DNA damage (40–43). In fact, multiple studies have shown the ability of low-dose radiation to stimulate DNA repair mechanisms, including in interventional cardiologists (29, 43).

The protective effect of radiation may be augmented or confounded by the lifestyle of the invasive cardiologists, since they are among the most physically active members of the profession, at least in the workplace. Most invasive cardiology procedures require protective attire and long hours of standing, which, by itself, may improve physical fitness. To prepare for the increased physical demands of the profession, it may be expected that invasive cardiologists are a healthcare population conscious of their physical fitness. Unfortunately, the physical fitness of the cardiologists was not assessed in the survey, and the literature is limited as well. Further study is required to determine the lifestyle variability among different types of cardiologists and its effect on their cardiovascular health.

## Limitations

The self-reported survey-based method of data collection is a major limitation; however, the fact that the population under investigation comprised board-certified health professionals, whose job it is to diagnose community members with the exact same conditions included in the survey, partially compensates for the self-reported format of this study. The alignment of risk factors of AA determined from this dataset with well-established risk factors provides additional confirmation regarding the accuracy of the dataset. Moreover, having cardiologists serve as both the low and high radiation exposure groups accounts for unknown confounding variables associated with the profession that cannot be adjusted by statistical analysis. An 8.8% response rate introduces the concern for selection bias and there is a high chance of responders and non-responders varying in subspeciality, level of experience and radiation exposure, and prevalence of AA due to personal interest in the study which need to be considered. However, the subspeciality response rate is similar in the responders compared to non-responders. Radiation exposure in this survey was measured in lifetime hours rather than actual dosage which is seldom tracked by physicians themselves. Over the years, there have been significant advancement in radiation protection so “lifetime radiation hours” may vary from “true radiation exposure hours” based on a cardiologist's years of practice. Nevertheless, the positive correlation of this measure with the risk of cataracts in this study population validates the methodology.

The cross-sectional de-identified nature of this study does not allow for follow-up with the participants to deduce the timeline of cataracts or AA as it pertains to the course of their radiation exposure. In addition, the amount of radiation exposure per procedure varies based on the institutional setting, the level of training, and the quality of or adherence to protective apparel. Physicians at academic institutions and fellows-in-training endure more radiation than their experienced, non-teaching counterparts, which should be considered when interpreting this data. Further study is merited to account for these intrinsic shortcomings (14).

## Conclusion

In this survey-based study of board-certified cardiologists, increased hours of radiation exposure was associated with decreased prevalence of AA, independent of conventional risk factors. This is a hypothesis generating study and a variety of theories can explain these paradoxical findings. Further long-term investigation is warranted in a larger and more detailed population to properly identify the factors that modify the risk of AA in cardiologists.

## Data Availability Statement

The raw data supporting the conclusions of this article will be made available by the authors, without undue reservation.

## Ethics Statement

The studies involving human participants were reviewed and approved by Louisiana State University Health Sciences Center Shreveport Institutional Review Board. Written informed consent for participation was not required for this study in accordance with the national legislation and the institutional requirements.

## Author Contributions

PD devised the study objective, study design, and the survey along with RT, CV, and SJ. Survey questions were revised based on critical input from SCha, SChu, RH, MB, PK, and SB. PD served as the principal investigator. RB performed the statistical analysis and aided in interpretation of the results. Acquisition and interpretation of data was performed by all authors. The manuscript was written by RT, CV, RB, and PD. Critical revision of the manuscript for important intellectual content was provided by SCha, SChu, RH, MB, PK, RG, BO, and SB. All authors contributed to the article and approved the submitted version.

## Funding

This study was funded by an institutional grant, for which C.G. Kevil is the institution's PI.

## Conflict of Interest

The authors declare that the research was conducted in the absence of any commercial or financial relationships that could be construed as a potential conflict of interest.

## Publisher's Note

All claims expressed in this article are solely those of the authors and do not necessarily represent those of their affiliated organizations, or those of the publisher, the editors and the reviewers. Any product that may be evaluated in this article, or claim that may be made by its manufacturer, is not guaranteed or endorsed by the publisher.

## Supplementary Material

Supplemental Material 1 Cardiovascular health of cardiologists.

Supplemental Figure 1 Graphical representation of participant recruitment to final study cohort. Participants were recruited through three professional cardiovascular societies. There was an 8.8% response rate among those who received the survey, of which, 1,033 participants were included in the final study (>50 years of age). Interventional cardiologists and electrophysiologists predominately had >10,000 h of radiation exposure while all other specialties predominately had ≤10,000 h of radiation exposure.

Supplemental Table 1 Prevalence of atrial arrhythmia in cardiologists based on characteristics (all respondents sample).

Supplemental Table 2 Prevalence of atrial arrhythmia in cardiologists performing each procedure.

Supplemental Table 3 Prevalence of comorbidity in cardiologists based on presence of atrial arrhythmia (*n* = 1,033).

Supplemental Table 4 Prevalence of atrial arrhythmia in cardiologists based on characteristics (>50 years of age respondents sample).
