# Supplementary material for: The Inverse Correlation Between the Duration of Lifetime Occupational Radiation Exposure and the Prevalence of Atrial Arrhythmia
Source: Front Cardiovasc Med. 2022 May 30;9:863939. doi: 10.3389/fcvm.2022.863939 (PMC9196104; doi:10.3389/fcvm.2022.863939)
Supplement: Supplemental Material 1 — Cardiovascular health of cardiologists. [file Data_Sheet_1.PDF]

Cardiovascular Health of Cardiologists

LOUISIANA STATE UNIVERSITY HEALTH SCIENCES CENTER in Shreveport Institutional Review Board (IRB) for the Protection of Human Research Subjects

CONSENT LETTER

Cardiovascular Health of Cardiologists

You are invited to participate in a study assessing the Cardiovascular Health of Cardiologists. We aim to learn the impact of radiation exposure in the Cardiovascular Health of Cardiologists stratified by subspecialties. You were selected as a possible participant in this study because you are a present or retired board-certified physician. You must be 18 years of age or older to participate.

The survey is designed to gather data about your demographics, occupational history and medical history. It will take approximately 5 minutes to complete. The survey is completely anonymous and no identifiable information will be collected. You will not directly benefit by completing the survey, but your responses will be used to help us increase cognizance about the impact of radiation exposure in the Cardiovascular Health of Cardiologists.

The risks of participating in this study are extremely limited. If you elect to complete the electronic survey on a mobile device, data charges may apply from your phone service provider. Please do not complete the survey while driving or operating machinery. The research team will not be held responsible for any data charges or injuries incurred while completing the survey. Please do not complete the survey while in a medical setting as it can affect patient care.

Your decision whether to participate will not influence your current or future relationships with your health institution LSUHSC-Shreveport or any organization in involved in this study. Answers to the survey will be accessible only to approved members of the research team based at LSU-Health Sciences Center in Shreveport, LA, LSU-Health Sciences Center in New Orleans, LA, and LSU-Pennington Biomedical Research Center in Baton Rouge, LA.

By completing the following survey, you acknowledge that you are a present or retired board-certified Cardiologist over the age of 18, agree that you understand the risk and benefits, and consent to be a participant in this survey. Please do not put your name on this form. No personal identifiers will be collected.

What about Confidentiality?

Efforts will be made to keep your personal information confidential. We cannot guarantee absolute confidentiality. Please feel free to ask questions regarding this study. You may contact Rithika Thirumal at rthiru@lsuhsc.edu with

your concerns.

You may also contact the Human Research Protection Program (which is a group of people who review the research to protect your rights) or the Institutional Official of LSUHSC-S at (318) 813-1350 (http://www.lsuhscshreveport.edu/Research/HRPP-Home/index) for:

additional help with any questions about the research

voicing concerns or complaints about the research

obtaining answers to questions about your rights as a research participant

concerns in the event the research staff could not be reached

the desire to talk to someone other than the research staff

If you decide to participate, please scroll down and complete the brief survey.

Thank you for your time.

Sincerely,

Paari Dominic, MD, LSU Health Shreveport – Center for Cardiovascular Diseases and Sciences/School of Medicine

Steven Bailey, MD, LSU Health Shreveport – Center for Cardiovascular Diseases and Sciences/School of Medicine

Pavan Katikaneni, MD, LSU Health Shreveport – Center for Cardiovascular Diseases and Sciences/School of Medicine

Lucio Miele, MD, PhD, LSU Health New Orleans – Department of Genetics/School of Medicine

Ronald Horswell, PhD, LSU Pennington Biomedical Research Center

San Chu, MS, MAPSST, LSU Pennington Biomedical Research Center

Angela Bennett, BS, LSU Health Shreveport – Center for Cardiovascular Diseases and Sciences

Sania Jiwani, MD, LSU Health Shreveport – Center for Cardiovascular Diseases and Sciences

Catherine Vanchiere, MD, LSU Health Shreveport – School of Medicine

Rithika Thirumal, LSU Health Shreveport – School of Medicine

Sex:	◯ Male<br>◯ Female<br>◯ Other
Age:	◯ >18<br>◯ 18-25<br>◯ 26-30<br>◯ 31-35<br>◯ 36-40<br>◯ 41-45<br>◯ 46-50<br>◯ 51-55<br>◯ 56-60<br>◯ 61-65<br>◯ 66-70<br>◯ 71-75<br>◯ 76 and above<br>(Please select the range that includes your current age)
Approximate Height:	◯ < 4' 6''<br>◯ 4' 6"<br>◯ 4' 7"<br>◯ 4' 8"<br>◯ 4' 9"<br>◯ 4' 10"<br>◯ 4' 11"<br>◯ 5'<br>◯ 5' 1"<br>◯ 5' 2"<br>◯ 5' 3"<br>◯ 5' 4"<br>◯ 5' 5"<br>◯ 5' 6"<br>◯ 5' 7"<br>◯ 5' 8"<br>◯ 5' 9"<br>◯ 5' 10"<br>◯ 5' 11"<br>◯ 6'<br>◯ 6' 1"<br>◯ 6' 2"<br>◯ 6' 3"<br>◯ 6' 4"<br>◯ 6' 5"<br>◯ 6' 6"<br>◯ 6' 7"<br>◯ 6' 8"<br>◯ 6' 9"<br>◯ 6' 10"<br>◯ 6' 11"<br>◯ 7'<br>◯ >7'

Approximate Weight (lbs):	◯ < 100<br>◯ 100<br>◯ 110<br>◯ 120<br>◯ 130<br>◯ 140<br>◯ 150<br>◯ 160<br>◯ 170<br>◯ 180<br>◯ 190<br>◯ 200<br>◯ 210<br>◯ 220<br>◯ 230<br>◯ 240<br>◯ 250<br>◯ 260<br>◯ 270<br>◯ 280<br>◯ 290<br>◯ 300<br>◯ 310<br>◯ 320<br>◯ 330<br>◯ 340<br>◯ 350<br>◯ 360<br>◯ 370<br>◯ 380<br>◯ 390<br>◯ 400<br>◯ > 400<br>(Please select the option that is closest to your weight)
Please select all of the following racial groups that you identify with:	☐ American Indian/Alaska Native<br>☐ Asian<br>☐ Black or African American<br>☐ Native Hawaiian or other Pacific Islander<br>☐ White or Caucasian<br>☐ Other
Ethnicity:	◯ Hispanic<br>◯ Non-Hispanic<br>◯ Unknown
Please select all specialties you are board-certified in:	☐ Interventional Cardiology<br>☐ Electrophysiology<br>☐ Echocardiology<br>☐ Heart Failure Cardiology<br>☐ Nuclear Imaging Cardiology<br>☐ Adult Cardiology<br>☐ Pediatric Cardiology<br>☐ Cardiac/Vascular Surgery<br>☐ Internal Medicine<br>☐ Other<br>(You must be a board-certified Physician to participate in this survey)
What "other" field of medicine do you specialize in?	__________________________________

Are you currently practicing medicine?

◯ Yes
◯ No/Retired

For how many years (currently or before retirement) have you practiced medicine?

◯ 1
◯ 2
◯ 3
◯ 4
◯ 5
◯ 6
◯ 7
◯ 8
◯ 9
◯ 10
◯ 11
◯ 12
◯ 13
◯ 14
◯ 15
◯ 16
◯ 17
◯ 18
◯ 19
◯ 20
◯ 21
◯ 22
◯ 23
◯ 24
◯ 25
◯ 26
◯ 27
◯ 28
◯ 29
◯ 30
◯ 31
◯ 32
◯ 33
◯ 34
◯ 35
◯ 36
◯ 37
◯ 38
◯ 39
◯ 40
◯ 41
◯ 42
◯ 43
◯ 44
◯ 45
◯ 46
◯ 47
◯ 48
◯ 49
◯ 50
◯ 51
◯ 52
◯ 53
◯ 54
◯ 55
◯ 56
◯ 57
◯ 58
◯ 59
◯ 60
◯ >60

On average, how many hours do you work per week?

- ◯ 5
- ◯ 10
- ◯ 15
- ◯ 20
- ◯ 25
- ◯ 30
- ◯ 35
- ◯ 40
- ◯ 45
- ◯ 50
- ◯ 55
- ◯ 60
- ◯ 65
- ◯ 70
- ◯ 75
- ◯ 80
- ◯ 85
- ◯ 90
- ◯ 95
- ◯ 100
- ◯ 105
- ◯ 110
- ◯ >110

(If retired, please answer based on when you practiced)

Please answer the following questions regarding occupational radiation exposure. If you are retired, answer based on when you were practicing.

Select all procedures you perform:

- ☐ Percutaneous Coronary Intervention
- ☐ Atherectomy
- ☐ Angioplasty
- ☐ Coronary Thrombectomy
- ☐ Transcatheter Aortic or Mitral Valve Replacement
- ☐ Valvuloplasty
- ☐ Repair of Congenital Heart Disease
- ☐ Electrophysiologic Studies
- ☐ Irregular Heart Rhythm Ablations
- ☐ Pacemaker/Defibrillator Placement
- ☐ Pacemaker/Defibrillator Lead Extraction
- ☐ Transthoracic Echocardiogram
- ☐ Transesophageal Echocardiogram
- ☐ Nuclear Imaging
- ☐ None of the above

Do you perform or assist in at least one procedure requiring fluoroscopy or cineangiography per week?

- ◯ Yes
- ◯ No

(If retired, please answer based on when you practiced)

Approximately how many hours are you exposed to fluoroscopy or cineangiography per week?

◯ 1
◯ 2
◯ 3
◯ 4
◯ 5
◯ 6
◯ 7
◯ 8
◯ 9
◯ 10
◯ 11
◯ 12
◯ 13
◯ 14
◯ 15
◯ 16
◯ 17
◯ 18
◯ 19
◯ 20
◯ 21
◯ 22
◯ 23
◯ 24
◯ 25
◯ 26
◯ 27
◯ 28
◯ 29
◯ 30
◯ 31
◯ 32
◯ 33
◯ 34
◯ 35
◯ 36
◯ 37
◯ 38
◯ 39
◯ 40
◯ 41
◯ 42
◯ 43
◯ 44
◯ 45
◯ 46
◯ 47
◯ 48
◯ 49
◯ 50
◯ 51
◯ 52
◯ 53
◯ 54
◯ 55
◯ 56
◯ 57
◯ 58
◯ 59
◯ 60
◯ 61
◯ 62
◯ 63
◯ 64
◯ 65
◯ 66
◯ 67
◯ 68
◯ 69

◯ 70
◯ 71
◯ 72
◯ 73
◯ 74
◯ 75
◯ 76
◯ 77
◯ 78
◯ 79
◯ 80
◯ >80

For how many years have you been performing or assisting in procedures requiring fluoroscopy or cineangiography?

- ◯ 1
- ◯ 2
- ◯ 3
- ◯ 4
- ◯ 5
- ◯ 6
- ◯ 7
- ◯ 8
- ◯ 9
- ◯ 10
- ◯ 11
- ◯ 12
- ◯ 13
- ◯ 14
- ◯ 15
- ◯ 16
- ◯ 17
- ◯ 18
- ◯ 19
- ◯ 20
- ◯ 21
- ◯ 22
- ◯ 23
- ◯ 24
- ◯ 25
- ◯ 26
- ◯ 27
- ◯ 28
- ◯ 29
- ◯ 30
- ◯ 31
- ◯ 32
- ◯ 33
- ◯ 34
- ◯ 35
- ◯ 36
- ◯ 37
- ◯ 38
- ◯ 39
- ◯ 40
- ◯ 41
- ◯ 42
- ◯ 43
- ◯ 44
- ◯ 45
- ◯ 46
- ◯ 47
- ◯ 48
- ◯ 49
- ◯ 50
- ◯ 51
- ◯ 52
- ◯ 53
- ◯ 54
- ◯ 55
- ◯ 56
- ◯ 57
- ◯ 58
- ◯ 59
- ◯ 60
- ◯ >60

(Please include years of Cardiology fellowships as well)

Please select all radiation-protective attire you are likely to wear while performing or assisting in procedures requiring fluoroscopy or cineangiography:	☐ Apron<br>☐ Vest<br>☐ Skirt<br>☐ Thyroid Collar<br>☐ Head Cap<br>☐ Glasses<br>☐ Shin Shields<br>☐ Leaded Gloves<br>☐ None of the above
How often do you wear the protective attire you selected above?	○ Always<br>○ Almost always<br>○ Sometimes<br>○ Almost never<br>○ Never
Do you use a protective shield/screen which stands between you and the fluoroscopic/angiographic machine?	○ Yes<br>○ No
What type of imaging do you use the most while performing procedures?	○ Pulsed Fluoroscopy<br>○ Low Frame Cineangiography<br>○ High Frame Cineangiography
Does your workplace monitor radiation exposure via dosimetry?	○ Yes<br>○ No
Does your institution have a threshold of radiation exposure above which you are not allowed to perform or assist in procedures anymore for a period of time?	○ Yes<br>○ No
Have you ever exceeded your monthly limit of radiation exposure and were not allowed to perform procedures for a period of time? If so, how many times has that occurred?	○ Never crossed the limit<br>○ 1<br>○ 2<br>○ 3<br>○ 4<br>○ 5<br>○ 6<br>○ 7<br>○ 8<br>○ 9<br>○ 10<br>○ 11<br>○ 12<br>○ 13<br>○ 14<br>○ 15<br>○ >15

Please answer the following questions about your personal medical history.

Have you received any CT scans, x-rays, radionuclide scans or other procedures requiring fluoroscopy or cineangiography for your personal medical care? If so, how many in your lifetime?	○ Never<br>○ 1-10 imaging in lifetime<br>○ more than 10 imaging
Have you ever received any Radiation Therapy for cancer treatment?	○ Yes<br>○ No

What type of Radiation Therapy did you receive?	◯ External Beam Radiation<br>◯ Internal Radiation (Brachytherapy)<br>◯ Unknown<br>◯ Other
Do you drink alcohol?	◯ Yes<br>◯ No<br>◯ Used to, but have quit
Approximately, how many drinks do you have per week?	◯ 1<br>◯ 2<br>◯ 3<br>◯ 4<br>◯ 5<br>◯ 6<br>◯ 7<br>◯ 8<br>◯ 9<br>◯ 10<br>◯ 11<br>◯ 12<br>◯ 13<br>◯ 14<br>◯ 15<br>◯ 16<br>◯ 17<br>◯ 18<br>◯ 19<br>◯ 20<br>◯ 21<br>◯ 22<br>◯ 23<br>◯ 24<br>◯ 25<br>◯ 26<br>◯ 27<br>◯ 28<br>◯ 29<br>◯ 30<br>◯ >30<br>(If quit drinking, please answer based on when you were drinking)
Do you use any tobacco products?	◯ Yes<br>◯ No<br>◯ Used to, but have quit
How many packs of cigarettes do you smoke per day currently?	◯ < 0.25<br>◯ 0.25<br>◯ 0.50<br>◯ 1<br>◯ 1.5<br>◯ 2<br>◯ 2.5<br>◯ 3<br>◯ >3<br>(If quit smoking, please answer based on when you were smoking)

How many years have you used tobacco products?

◯ 1
◯ 2
◯ 3
◯ 4
◯ 5
◯ 6
◯ 7
◯ 8
◯ 9
◯ 10
◯ 11
◯ 12
◯ 13
◯ 14
◯ 15
◯ 16
◯ 17
◯ 18
◯ 19
◯ 20
◯ 21
◯ 22
◯ 23
◯ 24
◯ 25
◯ 26
◯ 27
◯ 28
◯ 29
◯ 30
◯ 31
◯ 32
◯ 33
◯ 34
◯ 35
◯ 36
◯ 37
◯ 38
◯ 39
◯ 40
◯ 41
◯ 42
◯ 43
◯ 44
◯ 45
◯ 46
◯ 47
◯ 48
◯ 49
◯ 50
◯ 51
◯ 52
◯ 53
◯ 54
◯ 55
◯ 56
◯ 57
◯ 58
◯ 59
◯ 60
◯ >60

(If quit smoking, please answer based on when you were smoking)

How many years ago did you quit using cigarettes?	◯ 0-5 years ago<br>◯ 6-10 years ago<br>◯ 11-15 years ago<br>◯ 16-20 years ago<br>◯ 21-25 years ago<br>◯ More than 25 years ago
Please select all of the following diagnosis you have ever received:	☐ Aortic Atherosclerosis<br>☐ Cancer<br>☐ Cardiac Myxoma<br>☐ Cardiomyopathy<br>☐ Carotid Artery Disease<br>☐ Cataracts<br>☐ Chronic Obstructive Pulmonary Disease<br>☐ Congenital Heart Disease<br>☐ Congestive Heart Failure<br>☐ Coronary Artery Disease<br>☐ Constrictive Pericarditis<br>☐ Dermatitis<br>☐ Diabetes Mellitus<br>☐ Dyslipidemia<br>☐ Hypertension<br>☐ Infertility<br>☐ Ischemic Heart Disease<br>☐ Myocarditis<br>☐ Obstructive Sleep Apnea<br>☐ Peripheral Vascular Disease<br>☐ Pulmonary Fibrosis<br>☐ Pulmonary Hypertension<br>☐ Stroke<br>☐ TIA<br>☐ Thyroid Disease<br>☐ Valvular Heart Disease<br>☐ None of the above
Select the type of primary cancer you have had:	☐ Bone sarcoma<br>☐ Brain<br>☐ Breast<br>☐ Colon<br>☐ Endometrial<br>☐ Gastric<br>☐ Leukemia<br>☐ Lung<br>☐ Lymphoma<br>☐ Myelodysplastic syndrome<br>☐ Ovarian<br>☐ Pancreatic<br>☐ Prostate<br>☐ Skin<br>☐ Thyroid<br>☐ Testicular<br>☐ Other
Please specify the laterality of your breast malignancy:	◯ Right<br>◯ Left<br>◯ Bilateral
Please specify the laterality of your lung malignancy:	◯ Right<br>◯ Left<br>◯ Bilateral

What treatment did you receive for the cancer?	☐ Surgery<br>☐ Chemotherapy<br>☐ Radiotherapy<br>☐ None of the above
What is your New York Heart Association (NYHA) Classification for Congestive Heart Disease?	◯ Class I<br>◯ Class II<br>◯ Class III<br>◯ Class IV<br>◯ Unknown
Do you have Systolic or Diastolic Dysfunction? Or Both?	◯ Systolic Dysfunction<br>◯ Diastolic Dysfunction<br>◯ Both<br>◯ Unknown
On your latest Echocardiogram, what was the Ejection Fraction (EF)?	◯ Unknown<br>◯ 0%<br>◯ 5%<br>◯ 10%<br>◯ 15%<br>◯ 20%<br>◯ 25%<br>◯ 30%<br>◯ 35%<br>◯ 40%<br>◯ 45%<br>◯ 50%<br>◯ 55%<br>◯ 60%<br>◯ 65%<br>◯ 70%<br>◯ 75%<br>◯ 80%<br>◯ 85%<br>◯ 90%<br>◯ 95%<br>◯ 100%<br>(If percentage is in a range, select the lowest percentage (ex: 20-25%, select 20%))
For your Coronary Artery Disease, do you have any stents?	◯ Yes<br>◯ No<br>◯ Unknown

How many stents have you had placed for Coronary Artery Disease?	◯ Unknown<br>◯ 1<br>◯ 2<br>◯ 3<br>◯ 4<br>◯ 5<br>◯ 6<br>◯ 7<br>◯ 8<br>◯ 9<br>◯ 10<br>◯ 11<br>◯ 12<br>◯ 13<br>◯ 14<br>◯ 15<br>◯ >15
Do you have Type I or Type II Diabetes?	◯ Type I<br>◯ Type II
Are you on insulin for Diabetes?	◯ Yes<br>◯ No
Are you on any medical interventions for Obstructive Sleep Apnea currently?	◯ Yes, CPAP<br>◯ Yes, BiPAP<br>◯ Yes, Other treatment options<br>◯ No, I'm on no medical intervention
How often do you use the CPAP/BiPAP?	◯ Every night<br>◯ 5-6 nights per week<br>◯ 3-4 nights per week<br>◯ 1-2 nights per week<br>◯ < 3 times per month<br>◯ Never
What is the physiologic status of your thyroid disorder?	◯ Euthyroid<br>◯ Hypothyroid<br>◯ Hyperthyroid<br>(Please indicate the status at diagnosis, not after treatment.)
Have you undergone any of the following for your thyroid disorder? Please select all that apply	☐ Radioactive iodine uptake study<br>☐ Radioactive iodine ablation<br>☐ On oral medication for hyperthyroidism<br>☐ On oral medication for hypothyroidism<br>☐ None of the above
Which heart valve is diseased? Please select all that apply	☐ Aortic<br>☐ Mitral<br>☐ Pulmonic<br>☐ Tricuspid<br>☐ Unknown
Have you had a valvuloplasty or valve replacement?	◯ Yes<br>◯ No
Have you ever been diagnosed with an arrhythmia or abnormal heart rhythm?	◯ Yes<br>◯ No

What kind of arrhythmia(s) have you had? Select all that apply.	☐ Sinus Bradycardia<br>☐ Sinus Node Dysfunction<br>☐ Premature Atrial Contractions<br>☐ Atrial Tachycardia<br>☐ Atrioventricular Nodal Reentrant Tachycardia<br>☐ Atrioventricular Reentrant Tachycardia<br>☐ Atrial Flutter<br>☐ Atrial Fibrillation<br>☐ Multifocal Atrial Tachycardia<br>☐ Junctional Rhythm<br>☐ Junctional Tachycardia<br>☐ Premature Ventricular Contractions<br>☐ Ventricular Tachycardia<br>☐ Ventricular Fibrillation<br>☐ First Degree Heart Block<br>☐ Second Degree Heart Block<br>☐ Third Degree Heart Block<br>☐ Other
What type of atrial fibrillation do you currently have?	○ Paroxysmal<br>○ Persistent<br>○ Long-Standing Persistent<br>○ Permanent<br>○ Unknown
What was your age at the onset of atrial fibrillation?	○ < 10<br>○ 10-14<br>○ 15-19<br>○ 20-24<br>○ 25-29<br>○ 30-34<br>○ 35-39<br>○ 40-44<br>○ 45-59<br>○ 60-64<br>○ 65-69<br>○ 70-74<br>○ 75-79<br>○ 80-84<br>○ 85-89<br>○ 90-94<br>○ >=95
What medications do you use for atrial flutter or atrial fibrillation? Please select all that apply.	☐ Rate Control<br>☐ Rhythm Control<br>☐ Anticoagulation<br>☐ None

At what age did you start taking medication for atrial fibrillation/flutter?	◯ < 10<br>◯ 10-14<br>◯ 15-19<br>◯ 20-24<br>◯ 25-29<br>◯ 30-34<br>◯ 35-39<br>◯ 40-44<br>◯ 45-59<br>◯ 60-64<br>◯ 65-69<br>◯ 70-74<br>◯ 75-79<br>◯ 80-84<br>◯ 85-89<br>◯ 90-94<br>◯ >=95
Please select all anti-hypertensive/rate control medications you are currently taking:	☐ Amlodipine (Norvasc)<br>☐ Atenolol (Tenormin)<br>☐ Benazepril (Lotensin)<br>☐ Bisoprolol (Zebeta)<br>☐ Captopril (Capoten)<br>☐ Carvedilol (Coreg)<br>☐ Diltiazem hydrochloride (Cardizem CD, Dilacor XR)<br>☐ Digoxin (Lanoxin)<br>☐ Enalapril (Vasotec)<br>☐ Labetolol (Trandate)<br>☐ Lisinopril (Prinivil, Zestril)<br>☐ Losartan (Cozaar)<br>☐ Metoprolol succinate (Toprol-XL)<br>☐ Metoprolol tartrate (Lopressor)<br>☐ Nadolol (Corgard)<br>☐ Nebivolol (Bystolic)<br>☐ Nicardipine (Cardene)<br>☐ Nifedipine (Procardia)<br>☐ Olmesartan (Benicar)<br>☐ Pindolol (Visken)<br>☐ Propranolol (Inderal)<br>☐ Sacubitril/Valsartan (Entresto)<br>☐ Spironolactone (Aldactone)<br>☐ Valsartan (Diovan)<br>☐ Verapamil (Calan, Verelan)<br>☐ Other<br>☐ I do not take any anti-hypertensive/rate control medications<br>(Select medications even if taking for other conditions)

Please select all rhythm control medications you are currently taking:	☐ Amiodarone (Cordarone, Pacerone)<br>☐ Disopyramide (Norpace, Rythmodan)<br>☐ Dofetilide (Tikosyn)<br>☐ Dronedarone (Multaq)<br>☐ Flecainide (Tambocor)<br>☐ Mexiletine (Mexitil)<br>☐ Procainamide (Procan, Pronestyl)<br>☐ Propafenone (Rythmol)<br>☐ Quinidine (Quinidex)<br>☐ Sotalol (Betapace)<br>☐ Other<br>☐ I do not take any rhythm control medications<br>(Select medications even if taking for other conditions)
Please select all anticoagulation medications you are currently taking:	☐ Abciximab (ReoPro)<br>☐ Apixaban (Eliquis)<br>☐ Aspirin<br>☐ Clopidogrel (Plavix)<br>☐ Dabigratan (Pradaxa)<br>☐ Dipyrimadole Dipyrimadole/Aspirin (Aggrenox)<br>☐ Edoxaban (Savaysa)<br>☐ Enoxaparin (Lovenox)<br>☐ Eptifibatide (Integrilin)<br>☐ Fondaparinux (Arixtra)<br>☐ Heparin (Hemochron, Hep-Lock)<br>☐ Prasugrel (Effient)<br>☐ Rivaroxaban (Xarelto)<br>☐ Ticagrelor (Brilinta)<br>☐ Ticlodipine (Ticlid)<br>☐ Warfarin (Coumadin)<br>☐ Other<br>☐ I do not take any anticoagulation medications
What "other" anticoagulation medication(s) are you currently taking?	______________________________
Have you had a WATCHMAN device placement (closure of the left atrial appendage)?	○ Yes<br>○ No
Have you ever undergone cardioversion? If so, which type? Select all that apply:	☐ Pharmacologic Cardioversion<br>☐ Electrical Cardioversion<br>☐ Never been cardioverted
Have you undergone any EP (electrophysiologic) studies for the arrhythmia?	○ Yes<br>○ No
Have you undergone catheter ablation(s) for the arrhythmia?	○ Yes<br>○ No
How many catheter ablations have you undergone for the arrhythmia?	○ 1<br>○ 2<br>○ 3<br>○ 4<br>○ 5<br>○ 6<br>○ >7

Do you have a Pacemaker or an Automatic Implantable Cardioverter Defibrillator (AICD)?	☐ Pacemaker<br>☐ AICD<br>☐ Neither
What was the indication for pacemaker/AICD placement? Select all that apply	☐ AV Block<br>☐ Cardiac Resynchronization Therapy (Systolic Heart Failure)<br>☐ Chronic Bifascicular Block<br>☐ Hypersensitive Carotid Sinus Syndrome<br>☐ Hypertrophic Cardiomyopathy<br>☐ Long QT Syndrome<br>☐ Neurocardiogenic Syncope<br>☐ Primary Prevention for at those risk of VT/VF<br>☐ Secondary Prevention post VT/VF<br>☐ Sinus Node Dysfunction<br>☐ Other
