# Supplementary figures and images for: The Inverse Correlation Between the Duration of Lifetime Occupational Radiation Exposure and the Prevalence of Atrial Arrhythmia
Source: Front Cardiovasc Med. 2022 May 30;9:863939. doi: 10.3389/fcvm.2022.863939 (PMC9196104; doi:10.3389/fcvm.2022.863939)

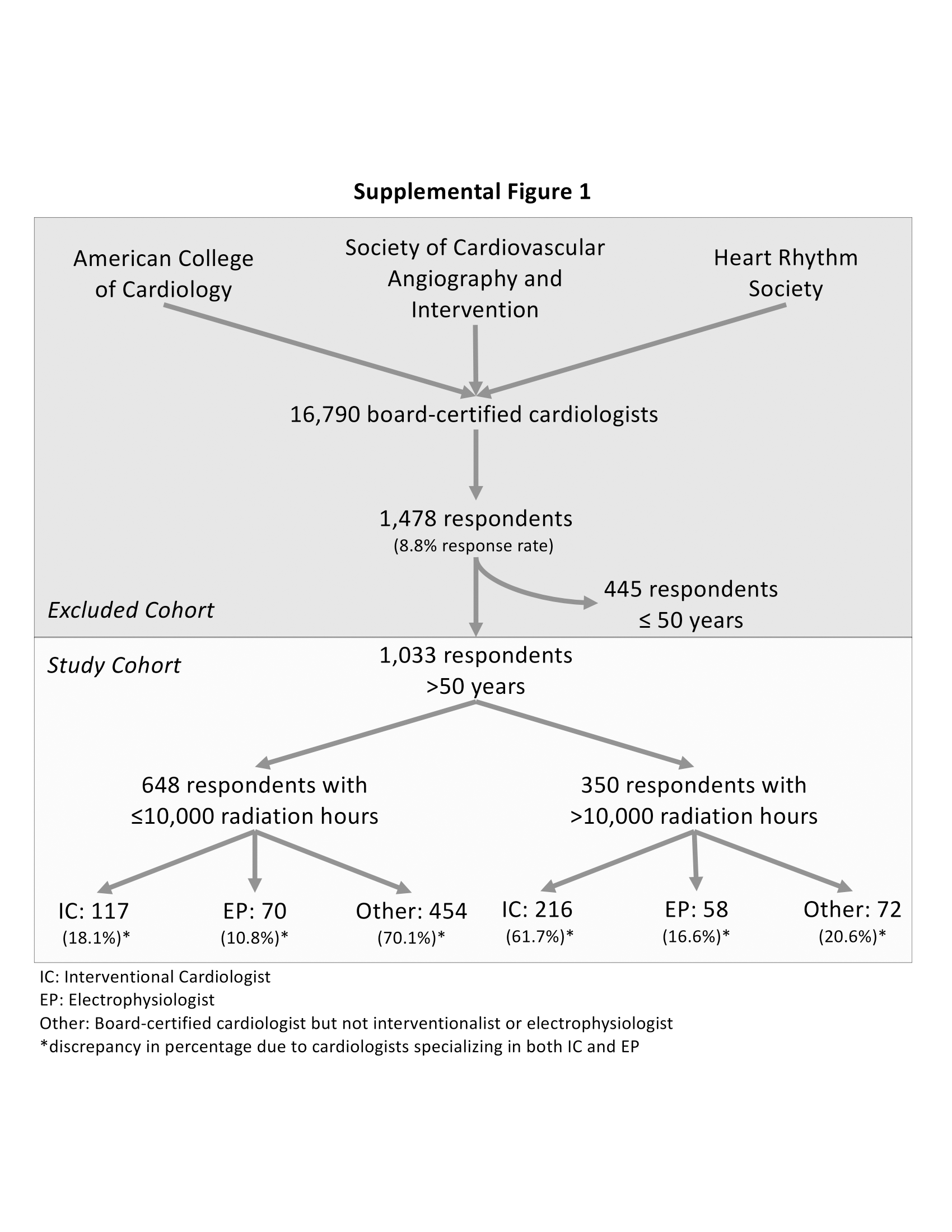

Supplement: Supplemental Figure 1 — Graphical representation of participant recruitment to final study cohort. Participants were recruited through three professional cardiovascular societies. There was an 8.8% response rate among those who received the survey, of which, 1,033 participants were included in the final study (>50 years of age). Interventional cardiologists and electrophysiologists predominately had >10,000 h of radiation exposure while all other specialties predominately had ≤10,000 h of radiation exposure. [file Image_1.TIFF]
