# Supplementary material for: The Inverse Correlation Between the Duration of Lifetime Occupational Radiation Exposure and the Prevalence of Atrial Arrhythmia
Source: Front Cardiovasc Med. 2022 May 30;9:863939. doi: 10.3389/fcvm.2022.863939 (PMC9196104; doi:10.3389/fcvm.2022.863939)
Supplement: Supplemental Table 1 — Prevalence of atrial arrhythmia in cardiologists based on characteristics (all respondents sample). [file Data_Sheet_2.pdf]

Supplemental Table 1. Prevalence of Atrial Arrhythmia in Cardiologists Based on Characteristics (All Respondents Sample)

		**# of Cardiologists (%) n = 1471***	**Prevalence of AA (%)**	***p*-value†**
**Demographic Characteristics**				
Sex	Male	1262 (86.1%)	156 (12.4%)	<0.001
	Female	204 (13.9%)	6 (2.9%)	
Age	≥ 66 years	490 (33.4%)	105 (21.4%)	<0.001
	≤ 65 years	979 (66.6%)	56 (5.7%)	
Race	White/Caucasian	1167 (79.0%)	148 (12.7%)	<0.001
	Black/African American	29 (2.0%)	2 (6.9%)	
	Other	269 (18.4%)	12 (4.5%)	
Ethnicity	Hispanic	36 (2.4%)	3 (8.3%)	0.569
	Non-Hispanic	1265 (85.6%)	144 (11.4%)	
**Occupational History**				
Type of Cardiologist	EP and Interventional Cardiology	757 (51.2%)	57 (7.5%)	<0.001
	Other	721 (48.8%)	107 (14.8%)	
Protective Attire worn	Head Cap	173 (11.7%)	8 (4.6%)	0.004
	Shin Shields	27 (1.8%)	3 (11.1%)	1.000
	Front Shield	915 (84.4%)	82 (9.0%)	<0.001
	Vest or Apron	1083 (73.3%)	115 (10.6%)	0.333
**Social History**				
Hx of Alcohol Use		1104 (74.7%)	134 (12.1%)	0.027
Hx of Alcohol Abuse		88 (6.0%)	15 (17.0%)	0.079
Hx of Smoking	Present, current	32 (2.2%)	7 (21.9%)	<0.001
	Absent, quit	92 (6.3%)	27 (29.3%)	
	Absent, never	1343 (91.5%)	124 (9.2%)	

*Sample number may vary due to exclusion of respondents electing to not answer

†Chi-squared test, $p<0.05$ is considered significant

Supplemental Table 2. Prevalence of Atrial Arrhythmia in Cardiologists Performing Each Procedure

**All Respondents Sample**	**# of Cardiologists (%) n = 1478**	**# of Cardiologists with AA (%)**	**Likelihood of AA Unadjusted OR (95% CI)[a]**	***p*-value[b]**
**Procedure**				
Atherectomy	469 (31.7%)	38 (8.1%)	0.63 (0.41 - 0.98)	0.038
Angiography	666 (45.1%)	76 (11.4%)	0.92 (0.64 - 1.34)	0.663
Coronary Thrombectomy	523 (35.4%)	45 (8.6%)	0.67 (0.44 - 1.02)	0.063
Valve Replacement	149 (10.1%)	10 (6.7%)	0.51 (0.25 - 1.04)	0.063
Valvuloplasty	232 (15.7%)	19 (8.2%)	0.64 (0.37 - 1.10)	0.108
Congenital Heart Disease Repair	115 (7.8%)	7 (6.1%)	0.46 (0.21 - 1.05)	0.065
Electrophysiologic Study	276 (18.7%)	19 (6.9%)	0.53 (0.31 - 0.91)	0.022
Irregular Rhythm Ablation	234 (15.8%)	15 (6.4%)	0.49 (0.27 - 0.89)	0.019
Pacemaker/Defibrillator Placement	497 (33.6%)	61 (12.3%)	1.00 (0.68 - 1.48)	1.000
Pacemaker/Defibrillator Lead Extraction	178 (12.0%)	10 (5.6%)	0.43 (0.21 - 0.86)	0.017
Percutaneous Angiography Intervention	652 (44.1%)	70 (10.7%)	0.86 (0.59 - 1.25)	0.433
None of the Above	440 (29.8%)	54 (12.0%)	Reference Category	

**>50 Years of Age Sample**	**# of Cardiologists (%) n = 1033**	**# of Cardiologists with AA (%)**	**Likelihood of AA Unadjusted OR (95% CI)[a]**	***p*-value[b]**
**Procedure**				
Atherectomy	309 (29.9%)	35 (11.9%)	0.66 (0.42 - 1.05)	0.077
Angiography	480 (46.5%)	69 (15.0%)	0.87 (0.58 - 1.28)	0.473
Coronary Thrombectomy	358 (34.7%)	42 (12.2%)	0.69 (0.44 - 1.06)	0.092
Valve Replacement	65 (6.3%)	7 (11.5%)	0.62 (0.27 - 1.44)	0.268
Valvuloplasty	139 (13.5%)	16 (11.9%)	0.67 (0.37 - 1.22)	0.193
Congenital Heart Disease Repair	63 (6.1%)	4 (6.6%)	0.35 (0.12 - 1.01)	0.051
Electrophysiologic Study	170 (16.5%)	18 (10.7%)	0.61 (0.34 - 1.08)	0.092
Irregular Rhythm Ablation	131 (12.7%)	14 (10.8%)	0.62 (0.33 - 1.16)	0.133
Pacemaker/Defibrillator Placement	360 (34.8%)	59 (16.9%)	1.01 (0.67 - 1.52)	0.959
Pacemaker/Defibrillator Lead Extraction	106 (10.3%)	10 (9.6%)	0.54 (0.26 - 1.10)	0.089
Percutaneous Angiography Intervention	474 (45.9%)	66 (14.6%)	0.83 (0.56 - 1.24)	0.371
None of the Above	314 (30.4%)	51 (16.2%)	Reference Category	

[a]Odds ratio with 95% confidence interval - Reference: None of the above

[b]$p<0.05$ is considered significant

Supplemental Table 3. Prevalence of comorbidity in cardiologists based on presence of atrial arrhythmia (n = 1033)

	**All Responses**	**>50 Years of Age**			
	**Prevalence (%) n = 1478[a]**	**Prevalence (%) n = 1033[a]**	**Prevalence of Comorbidity with AA (%) n = 152[a]**	**Prevalence of Comorbidity without AA (%) n = 881[a]**	***p*-value[b]**
**Comorbidities**					
Atrial Arrhythmia	164 (11.1%)	147 (14.7%)			
Aortic Atherosclerosis	40 (2.7%)	40 (3.9%)	16 (10.5%)	24 (2.7%)	<0.001
Cancer	167 (11.3%)	157 (15.2%)	29 (19.1%)	128 (14.5%)	0.149
Cardiomyopathy	11 (0.7%)	11 (1.1%)	7 (4.6%)	4 (0.5%)	<0.001
Carotid Artery Disease	20 (1.4%)	20 (1.9%)	7 (4.6%)	13 (1.5%)	0.019
Cataracts	267 (18.1%)	262 (25.4%)	50 (32.9%)	212 (24.1%)	0.021
Chronic Obstructive Pulmonary Disease	13 (0.9%)	12 (1.2%)	5 (3.3%)	7 (0.8%)	0.021
Congestive Heart Failure	11 (0.7%)	11 (1.1%)	7 (4.6%)	4 (0.5%)	<0.001
Coronary Artery Disease	129 (8.7%)	128 (12.4%)	45 (29.6%)	83 (9.4%)	<0.001
Dermatitis	44 (3.0%)	37 (3.6%)	2 (1.3%)	35 (4.0%)	0.104
Diabetes Mellitus	82 (5.5%)	73 (7.1%)	18 (11.8%)	55 (6.2%)	0.013
Dyslipidemia	400 (27.1%)	352 (34.1%)	56 (36.8%)	296 (33.6%)	0.436
Hypertension	450 (30.4%)	407 (39.4%)	70 (46.1%)	337 (38.3%)	0.069
Infertility	35 (2.4%)	21 (2.0%)	3 (2.0%)	18 (2.0%)	1.000
Ischemic Heart Disease	50 (3.4%)	50 (4.8%)	18 (11.8%)	32 (3.6%)	<0.001
Myocarditis	3 (0.2%)	3 (0.3%)	2 (1.3%)	1 (0.1%)	0.058
Obstructive Sleep Apnea	118 (8.0%)	108 (10.5%)	30 (19.7)	78 (8.9)	<0.001
Peripheral Vascular Disease	12 (0.8%)	11 (1.1%)	3 (2.0%)	8 (0.9%)	0.212
Pulmonary Fibrosis	4 (0.3%)	4 (0.4%)	3 (2.0%)	1 (0.1%)	0.011
Pulmonary Hypertension	4 (0.3%)	4 (0.4%)	2 (1.3%)	2 (0.2%)	0.105
Stroke/Transient Ischemic Attack	37 (2.5%)	34 (3.3%)	9 (5.9%)	25 (2.8%)	0.049
Thyroid Disease	95 (6.4%)	76 (7.4%)	15 (9.9%)	61 (6.9%)	0.199
Valvular Heart Disease	46 (3.1%)	43 (4.2%)	16 (10.5%)	27 (3.1%)	<0.001

[a]Sample number may vary due to exclusion of respondents electing to not answer

[b]Chi-squared test, $p<0.05$ is considered significant

Supplemental Table 4. Prevalence of Atrial Arrhythmia in Cardiologists Based on Characteristics (>50 Years of Age Respondents Sample)

		# of Cardiologists (%) n = 1033*	Prevalence of AA (%)	*p*-value†
**Demographic Characteristics**				
Sex	Male	915 (88.6%)	144 (15.7%)	0.004
	Female	111 (10.7%)	6 (5.4%)	
Age	≥ 66 years	490 (47.4%)	105 (21.4%)	<0.001
	≤ 65 years	543 (52.6%)	47 (8.7%)	
Race	White/Caucasian	903 (87.4%)	138 (15.3%)	0.379
	Black/African American	17 (1.6%)	2 (11.8%)	
	Other	106 (10.3%)	11 (10.4%)	
Ethnicity	Hispanic	29 (2.8%)	2 (6.9%)	0.294
	Non-Hispanic	876 (84.8%)	133 (15.2%)	
**Occupational History**				
Type of Cardiologist	EP and Interventional Cardiology	487 (47.1%)	51 (10.5%)	<0.001
	Other	546 (52.9%)	101 (18.5%)	
Protective Attire worn	Head Cap	85 (8.2%)	7 (8.2%)	0.078
	Shin Shields	18 (1.7%)	2 (11.1%)	1.000
	Front Shield	609 (59.0%)	75 (12.3%)	0.003
	Vest or Apron	744 (72.0%)	105 (14.1%)	0.381
**Social History**				
Hx of Alcohol Use		791 (76.6%)	123 (15.5%)	0.137
Hx of Alcohol Abuse		63 (6.2%)	14 (22.2%)	0.097
Hx of Smoking	Present, current	23 (2.2%)	7 (30.4%)	<0.001
	Absent, quit	919 (89.0%)	115 (12.5%)	
	Absent, never	81 (7.8%)	24 (29.6%)	

*Sample number may vary due to exclusion of respondents electing to not answer

†Chi-squared test, $p<0.05$ is considered significant
